# Low-tech, high impact: care for premature neonates in a district hospital in Burundi. A way forward to decrease neonatal mortality

**DOI:** 10.1186/s13104-015-1666-y

**Published:** 2016-01-16

**Authors:** Brigitte Ndelema, Rafael Van den Bergh, Marcel Manzi, Wilma van den Boogaard, Rose J. Kosgei, Isabel Zuniga, Manirampa Juvenal, Anthony Reid

**Affiliations:** Médecins Sans Frontières, BP 26, Bujumbura, Burundi; Operational Research Unit, Médecins Sans Frontières, Operational Centre Brussels, MSF-Luxembourg, Luxembourg, Luxembourg; Department of Obstetrics and Gynaecology, University of Nairobi, Nairobi, Kenya; Burundi Ministry of Health, District Hospital, Kabezi, Burundi

**Keywords:** Prematurity, Preterm, Operational research, District hospital, Burundi

## Abstract

**Background:**

Death among premature neonates contributes significantly to neonatal mortality which in turn represents approximately 40 % of paediatric mortality. Care for premature neonates is usually provided at the tertiary care level, and premature infants in rural areas often remain bereft of care. Here, we describe the characteristics and outcomes of premature neonates admitted to neonatal services in a district hospital in rural Burundi that also provided comprehensive emergency obstetric care. These services included a Neonatal Intensive Care Unit (NICU) and Kangaroo Mother Care (KMC) ward, and did not rely on high-tech interventions or specialist medical staff.

**Methods:**

A retrospective descriptive study, using routine programme data of neonates (born at <32 weeks and 32–36 weeks of gestation), admitted to the NICU and/or KMC at Kabezi District Hospital.

**Results:**

437 premature babies were admitted to the neonatal services; of these, 134 (31 %) were born at <32 weeks, and 236 (54 %) at 32–36 weeks. There were 67 (15 %) with an unknown gestational age but with a clinical diagnosis of prematurity. Survival rates at hospital discharge were 62 % for the <32 weeks and 87 % for the 32–36 weeks groups; compared to respectively 30 and 50 % in the literature on neonates in low- and middle-income countries. Cause of death was categorised, non-specifically, as “Conditions associated with prematurity/low birth weight” for 90 % of the <32 weeks and 40 % of the 32–36 weeks of gestation groups.

**Conclusions:**

Our study shows for the first time that providing neonatal care for premature babies is feasible at a district level in a resource-limited setting in Africa. High survival rates were observed, even in the absence of high-tech equipment or specialist neonatal physician staff. We suggest that these results were achieved through staff training, standardised protocols, simple but essential equipment, provision of complementary NICU and KMC units, and integration of the neonatal services with emergency obstetric care. This approach has the potential to considerably reduce overall neonatal mortality.

## Background

Prematurity, defined as birth at less than 37 completed weeks of gestation, affects 13 million babies globally, with 60 % of cases occurring in South Asia and sub-Saharan Africa [1, 2]. Complications of prematurity are the single most common cause of neonatal deaths (death occurring within the first 28 days of life). Globally, neonatal deaths account for more than 40 % of under-five mortality [3].

In developing countries, one of the contributing factors to high neonatal mortality rates, in particular among premature infants, is the lack of Neonatal Intensive Care Units (NICUs) at the level of district hospitals where most comprehensive obstetrics care takes place. In most of these countries, NICUs are limited to tertiary referral hospitals and remain inaccessible to rural communities. Even where NICUs are available, the case load of neonates requiring care may exceed the available bed capacity. To address the lack of neonatal facilities at the district level, a recent publication, “Born Too Soon”, recommends that care of premature infants should be managed in decentralized settings, and recognizes the need for research to adapt high-tech approaches to resource-limited contexts [4].

Burundi is such a context, with a high neonatal mortality rate of 42 deaths per 1000 live births in 2010, though this rate was not stratified for prematurity [2]. The high neonatal mortality rate in Burundi is one of the major barriers to sustainably achieving the fourth Millennium Development Goal of reducing the under-five mortality rate of 1990 by 2015 [5, 6].

In view of this high mortality rate, Médecins Sans Frontières (MSF), in collaboration with the Burundian Ministry of Health, started an NICU in Kabezi District. This unit was unique because—unlike other NICUs—it was part of a district hospital, rather than a tertiary referral hospital. The NICU was integrated into the Emergency Obstetric Care (EOC) package, and provided care for sick neonates using relatively simple equipment and without specialized neonatal consultants. This model was described in a recent publication which confirmed the possibility of providing neonatal care at the district level [7]. However, while recognizing the considerable contribution of prematurity to overall neonatal morbidity and mortality, this study did not examine prematurity in any detail. Furthermore, a review of the literature regarding premature neonates in sub-Saharan Africa shows only outcomes for the longer term (more than 1 year) and there are no studies examining immediate outcomes of neonatal care for premature infants in a district hospital setting [1].

In order to address this knowledge gap, and to better understand the determinants of this vulnerable population, this study was conducted to describe characteristics and treatment outcomes of premature neonates at Kabezi District Hospital, Burundi, between 2011 and 2012.

## Methods

### Design

This was a descriptive study involving a retrospective review of routinely collected hospital data.

### General setting

Burundi is a small, densely populated country of 8.6 million people, of whom 14 % are children under the age of 5 years [2, 8]. It is located in Central Africa, surrounded by Rwanda, Tanzania and the Democratic Republic of Congo. Government spending on health is approximately 21 US dollars per capita per year [8].

There are three tertiary care public hospitals and one private hospital in Burundi with NICUs, but district hospitals do not offer this kind of service because of lack of the necessary materials, adequate infrastructure and trained staff. Thus, access to NICU care for premature infants is very limited.

### Study site

Given the high rate of maternal and neonatal mortality in Burundi, and the humanitarian needs following armed conflicts that traumatized the country, MSF opened the EOC project in Kabezi district in 2006. The project has a catchment area of three districts (Kabezi, Isale and Rushubi) in the province of Rural Bujumbura, and represents approximately 186,000 habitants. The EOC project, part of Kabezi District Hospital and located 2 km from the hospital proper, was established as a referral centre for women with obstetrical complications in the district and does not providing primary obstetric and neonatal care (which is provided in Kabezi District Hospital itself). The details of the EOC component have been described elsewhere and include care for women with obstetric complications such as pre-eclampsia, prolonged or obstructed labour, uterine rupture, obstetric haemorrhage, and severe malaria [9]. Due to the referral pattern of cases, by 2008, the number of newborns who required special neonatal care had increased significantly, and an internal audit showed a high intra-hospital mortality rate. To address this need, a neonatal unit was set up in 2009 and integrated with the obstetric care programme.

The neonatal service consists of two wards. One ward is the NICU, with 17 beds, staffed by skilled and trained non-specialist personnel (a generalist doctor, a general paediatrician who is shared with the obstetrics centre, two nurses and two lactation assistants), uses specially developed standardised treatment protocols (including on-site training on their use), and is equipped with relatively low-technology equipment such as heated mattresses, electric pumps to administer milk via nasogastric tubes, intravenous supplies, and oxygen concentrators. There are no ventilators. These measures are described in detail in a previous study done in 2013 [7]. The second ward is the Kangaroo Mother Care (KMC), which has five beds, providing skin-to-skin warming and is supervised by a neonatal nurse and one lactation assistant. The components of KMC are skin-to-skin contact and promotion of breastfeeding to aid mother-infant bonding and provide warmth after birth, particularly for premature babies [10–12].

Neonates with the following complications are admitted to the NICU ward: neonatal sepsis, birth asphyxia, respiratory distress not otherwise specified, congenital malformations, and conditions associated with low birth weight such as hypoglycaemia, hypothermia, respiratory distress or apnea linked to prematurity. Definitions of these diagnoses are discussed in detail in a previous publication—diagnoses were made clinically, as only limited laboratory tests were available [7]. Premature and low birth weight neonates, especially those under 2000 grams, who are not sick, but need support with feeding, control of blood sugar, or extra warmth, are admitted directly to the KMC ward.

### Study population

All neonates born at less than 37 weeks gestation and admitted to the NICU or KMC ward at Kabezi district hospital during 2011 and 2012 were included in the study.

### Sources of data and data validation

Data were single-entered from patient case files into a structured EpiData database (v3.1, EpiData Association, Odense, Denmark). This database was checked against the routine project database (Excel based). It was implemented in 2011 to record all project data, automatically calculate indicators set by the project and prepare various reports. Data collection was managed by the project data manager, who checked for missing information with the medical team. Validation of the data was done by the medical director, the medical coordinator and epidemiologist of the MSF mission.

Gestational age was determined using the date of the last menstrual period. However, as most mothers could not accurately recollect the date of their last menstruation, ultrasound was routinely performed to confirm gestational age, in particular for mothers at risk of premature delivery, where accurate assessment the gestational age for e.g. lung maturation is of primordial importance. For patients who arrived with an imminent birth and for whom ultrasound was no longer possible, the age of the premature neonates was determined through clinical examination (Table 1).

**Table 1:** Criteria for rapid gestational assessment at delivery

Feature	36 Weeks and earlier	37–38 Weeks	39 Weeks & Beyond
Creases in soles of feet	1 or 2 transverse creases; posterior ¾ of sole smooth	Multiple creases; anterior 2/3 of heel smooth	Entire sole, including heel, covered with creases
Breast nodule^a^	2 mm	4 mm	7 mm
Scalp hair	Fine & woolly; fuzzy	Fine & woolly; fuzzy	Coarse & silky; each hair single-stranded
Ear lobe	No cartilage	Moderate amount of cartilage	Stiff ear lobe with thick cartilage
Testes & scrotum	Testes partially descended; scrotum small, with few rugae		Testes fully descended; scrotum normal size, with prominent rugae

^a^The breast nodule is not palpable before 33 weeks. Underweight full-term infants may have retarded breast development (Clinical significance of gestational age and objective measurement. *Pediatr Clin North Am*)

### Statistics and analysis

Simple summary statistics were generated using EpiData Analysis software (v.2.2.1.178, EpiData Association). Neonates were classified as <32 weeks (very preterm neonates) or 32–36 completed weeks (moderately preterm) of gestation, since this cut-off has been shown to affect outcomes [1]. Neonate Apgar scores at 5 min were categorized as (0–6) or (7–10), as described in other studies [7, 13]. Proportions between the different gestational age groups were compared using Chi square test.

### Ethics

This study was approved by the Burundi Ethics Review Committee and met the Médecins Sans Frontières’ (Geneva, Switzerland) Ethics Review Board-approved criteria for analysis of routinely-collected programme data. It also satisfied the requirements of the Ethics Advisory Group of the International Union against Tuberculosis and Lung Disease, Paris, France and met their approval.

As an analysis of routinely-collected data, consent was not obtained from the participants. But it met the six criteria for studies of routinely-collected data from the MSF ERB.

## Results

During 2011 and 2012, out of 4260 babies born in Kabezi District Hospital, 994 neonates were admitted to the neonatal unit services (NICU and/or KMC). Of these, 437 (44 %) were babies who were born prematurely. For 370 (85 %), a gestational age was known, while for 67 (15 %) the diagnosis of prematurity was made on clinical assessment alone, without an estimated gestational age, and they were excluded from the comparisons below. However, their discharge status was included. Of the 370 included premature neonates, 343 (93 %) were admitted to the NICU, followed by a stay in the KMC, and 27 (7 %) were admitted only to the KMC.

Characteristics of premature neonates are described in Table 2. Neonates of <32 weeks of gestation had lower birth weights and more active resuscitation than infants 32–36 weeks gestation. Both groups had relatively high caesarean section rates (34 and 43 % respectively), and no instrumental deliveries were performed in the <32 weeks group. Almost all mothers of premature neonates had antenatal maternal complications (mainly prolonged/obstructed labour and antepartum haemorrhage), and dexamethasone (for pulmonary maturation) was provided in 52 % (<32 weeks) and 40 % (32–36 weeks) of the cases.

**Table 2:** Characteristics of premature infants born at less than 37 weeks of gestation admitted to NICU and KMC wards at Kabezi District, Burundi (2011–2012)

Premature infants	<32 weeks of gestation N = 134 (%)	32–36 weeks of gestation N = 236 (%)	p-value
Birth weight (g)			
<1000	17 (13)	1 (0.4)	<0.0001
1000–1499	61 (46)	33 (14)	
1500–2499	47 (35)	181 (77)	
>2500	4 (3)	14 (6)	
Not recorded	5 (4)	7 (3)	
Sex			
Male	72 (54)	115 (49)	0.6
Female	61 (46)	117 (50)	
Not recorded	1 (1)	4 (2)	
APGAR score at 5 minutes			
0–6	54 (40)	71 (30)	0.1
7–10	74 (55)	151 (64)	
Not recorded	6 (5)	14 (6)	
Mode of delivery			
Caesarean section	46 (34)	101 (43)	0.06
Instrumental vaginal	0	4 (2)	
Non-instrumental vaginal	88 (66)	128 (54)	
Not recorded	0	3 (1)	
Perinatal interventions			
Steroid treatment^a^	69 (52)	94 (40)	0.06
Tocolytics used	58 (43)	66 (28)	0.01
Active birth resuscitation	107 (80)	151 (64)	0.005
Antenatal maternal complications			
Prolonged/obstructed labour	39 (29)	81 (34)	0.1
Ante-partum haemorrhage	20 (15)	25 (11)	
Sepsis	7 (5)	8 (3)	
(Pre-)eclampsia	1 (1)	13 (6)	
Uterine rupture	0	1 (0.4)	
Other severe conditions	57 (43)	81 (34)	

*NICU* Neonatal Intensive Care Unit, *KMC* Kangaroo Maternal Care

^a^Dexamethasone for lung maturation

Neonatal admission diagnoses are presented in Table 3. Given limited diagnostic facilities and lack of neonatal specialists, few specific diagnoses were recorded. The vast majority were categorised as “Other conditions linked to prematurity/low birth weight”.

**Table 3:** Admission diagnoses of premature infants born less than 37 weeks of gestation admitted in NICU and KMC wards at Kabezi District, Burundi (2011–2012)

Admission diagnosis	<32 weeks of gestation N = 134 (%)	32–36 weeks of gestation N = 236 (%)	p-value
Other conditions linked to prematurity/LBW^a^:			0.08
Very low birth weight (< 1500 g) Other	61 (46)	29 (12)	
	38 (28)	115 (49)	
Severe neonatal infections	12 (9)	33 (14)	
Perinatal asphyxia	10 (8)	39 (17)	
Congenital malformations	4 (3)	6 (3)	
Necrotizing enterocolitis	3 (2)	4 (2)	
Neonatal tetanus	1 (1)	1 (0.4)	
Other neonatal diseases	5 (4)	5 (2)	
No diagnosis recorded	0	4 (2)	

*NICU* Neonatal Intensive Care Unit, *KMC* Kangaroo Maternal Care, *LBW* low birth weight

^a^Conditions associated with low birth weight: such as hypoglycemia, hypothermia, respiratory distress or apnea linked to premature birth and not explained by other diagnoses Note: systematic glucose testing done in low birth weight neonates [10]

Table 4 shows discharge outcomes. Discharge survival rates were 62 % for neonates <32 weeks gestation and 87 % for neonates of 32–36 weeks gestation. In the first 24 h of life, 24 and 28 % of deaths occurred in the two groups respectively. The precise cause of neonatal death could rarely be established. For most neonates (90 % of the <32 weeks and 40 % of the 32–36 weeks of gestation group), the cause of death was recorded unspecifically as “related to prematurity or low birth weight”.

**Table 4:** Discharged outcomes and causes of death of premature infants at Kabezi District, Burundi (2011–2012)

	<32 weeks of gestation N = 134 (%)	32–36 weeks of gestation N = 236 (%)	p-value
Length of stay (days) − median (IQR)	11 (5–22)	9 (4–16)	0.04^a^
Discharge outcome			
Discharged	83 (62)	205 (87)	<0.0001
Died	41 (31)	25 (11)	
Transferred out	7 (5)	2 (1)	
Not recorded	3 (2)	4 (2)	
Time of death			
<24 hours	10 (24)	7 (28)	0.6
24–48 hours	6 (15)	2 (8)	
3–7 days	14 (34)	12 (48)	
8–28 days	10 (24)	4 (16)	
>28 days	1 (2)	0	

^a^Kruskal-Wallis test; *IQR* interquartile range

**Fig. 1:**
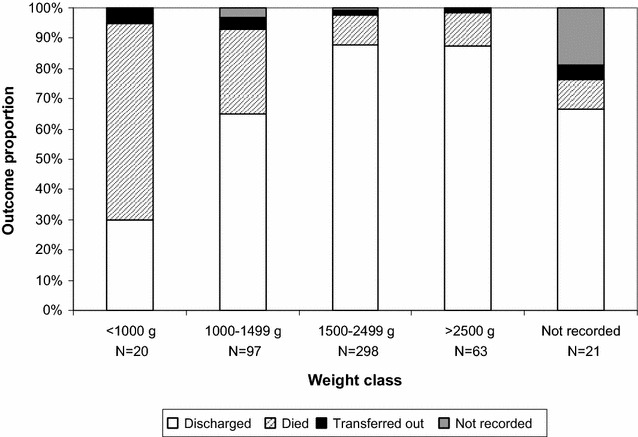
Outcomes stratified by weight class, for premature infants at Kabezi District, Burundi (2011–2012)

When broken down by weight, survival was related to birth weight. However, even among the lowest birth weight class (<1000 g) a survival rate of 30 % was observed (Fig. 1). Among the 67 neonates excluded from full analysis, as a result of their missing gestational age, survival rates were similar to the 32–36 weeks group, with 57 (85 %) discharged alive, eight (12 %) deaths, one (2 %) transferred out and one (2 %) undocumented outcome. In this group, only three neonates had a birth weight <1500 g, all of whom died, suggesting it matched more closely with the 32–36 weeks gestational age class.

## Discussion

This is one of the first studies reporting hospital outcomes of premature babies in a district hospital in sub-Saharan Africa. It describes how a district hospital providing neonatal services achieved high survival rates among neonates born prematurely, with more than 60 % of the neonates of gestational age <32 weeks and more than 80 % of gestational age 32–36 weeks discharged alive from hospital care. These results were achieved with basic, low-tech equipment and without specialist neonatal physician staff.

The strengths of this study were that, due to the high number of births at Kabezi District Hospital, a relatively large sample size of premature infants was achieved, and most data were captured in a systematic way. Additionally, as no neonates were lost to follow up and only a few were referred out of the hospital, the study included essentially the complete population of premature infants cared for in the hospital. In addition, the study adhered to STROBE guidelines for reporting on observational studies [14].

The study was, however, limited in several ways. Data on gestational age were missing for 15 % of the population, necessitating a clinical diagnosis of prematurity without age assessment; these patients were not included in the detailed analysis, but were reported on in terms of outcomes. This data gap was also flagged recently in a review of premature births [15]. Likewise, accurate diagnostic information of neonatal mortality was seriously lacking, with most neonates categorized in the non-specific “Conditions linked to prematurity/low birth weight” group. This concern was noted in our previous study on all neonates admitted to the hospital [7]. However, the diagnostic gap was even more marked in this study due to its focus on premature neonates, highlighting the challenge of accurate diagnosis in this population. In response, lists of standardized diagnoses, with clear case definitions have been implemented in the hospital over the course of 2012, potentially improving this situation. Finally, we were only able to report on hospital discharge outcomes, and were unaware of the long-term outcomes of premature neonates discharged from the hospital. This requires further follow-up studies at the community level.

A recent major review of the Born Too Soon Preterm Birth Action Group, reported on estimated survival rates of premature neonates in high- and low/middle-income countries [15]. This review of 15 million premature births reported a survival rate of approximately 50 % among neonates born around 34 weeks of gestation, and 30 % among neonates born at <32 weeks of gestation in low-income settings [15, 16]. Other studies have described similar survival rates (e.g. respectively 68 and 19 % in Malawi), and/or high risk ratios for neonates <32 weeks of gestation (pooled risk ratio of 25.8 in the African region) [17, 18]. The neonatal services in Kabezi District Hospital outperformed these published rates, pointing towards a strong programmatic approach.

We believe there are a number of programme features that produced these results, First, premature babies were treated immediately after birth at Kabezi District Hospital, as emergency obstetric care and neonatal services were well-integrated. Despite this being an EOC referral facility, a reasonable coverage (more than half in the <32 weeks group) with antenatal steroids was achieved, which may have had a positive impact on reducing mortality among premature neonates—due to the late presentation of many mothers, including those at risk of premature birth, increasing the rates of steroid coverage was challenging [4]. Since all the mothers were referred for complications, the newborns did not have to be transferred into another specialized health facility. We suspect that delays in reaching specialized neonatal services add considerably to the high mortality in these babies in other contexts.

Second, the neonatal services consisted of integrated NICU and KMC wards—all neonates passed through the KMC during their hospital stay, an approach that has been shown to substantially reduce neonatal morbidity and mortality in hospital settings and that has been recommended to be rolled out in low-income settings [19].

Third, within the programme itself, a strong focus was placed on training and implementation of standardized protocols for neonatal care, with specific adaptations for premature infants (Table 5). Thus, the care could usually be carried out by nursing staff following protocols and did not rely on highly-trained physicians.

**Table 5:** NICU and KMC services provided at CURGO in Kabezi District

Neonatology ward^†^	KMC ward
Care for sick neonates, regardless of weight and non-sick LBW neonates <1250 g until stabilisation^‡^	Care for non-sick, LBW neonates
17 beds, decreased to 12 by February 2012	5 beds shared with mothers
Daytime staff: 2 dedicated nurses; 1 supervisor	Daytime staff: 1 dedicated nurse;
2 lactation assistants (shared);	2 lactation assistants (shared);
1 doctor (shared)	1 doctor (shared)
1 General Pediatrician	
Nighttime staff: 2 dedicated nurses; 1 doctor on call (shared)	Nighttime staff: no dedicated staff; nurse from neonatology ward covers the KMC ward; 1 doctor on call (shared)
Protocols	Protocols
Basic warming equipment (3 heating mattress since june 2011)	Skin-to-skin care
Oxygen concentrators	Breastfeeding support Nasogastric tube feeding or alternative feeding techniques if needed
Electric pumps	
Intravenous fluids	Bedside monitoring of blood glucose, weight and temperature
Intravenous antibiotics (ampicillin, gentamicin, cefotaxime, cloxacillin, metronidazole)	Oral caffeine (to prevent apnoeaof prematurity)
	Oral ferrous acid folic (to prevent anemia)
Nasogastric tube feeding if needed bedside monitoring of blood glucose, haemoglobin, and oxygen saturation blood transfusion	

A laboratory at CURGO performs basic tests (i.e., white blood cell count, malaria microscopy). Culture, bilirubin, C-reactive protein, blood gases and electrolytes are not performed

*KMC* Kangaroo Mother Care, *LBW* low birth weight, *CURGO* Centre d’Urgences Gynéco Obstetricales

^†^Services not available in the neonatology ward included blood culture, mechanical ventilation, phototherapy, incubators and on-site surgical and radiology facilities

^‡^Neonates with birth weight <1250 g were cared for in the neonatology ward until stabilisation, as they usually required intravenous fluids, intravenous medications and possibly oxygen initially. These were not available in the KMC ward

A programmatic weakness which remained, however, was the impossibility of performing special clinical tests to determine causes of illness, such as C-reactive protein testing, analysis of cerebrospinal fluid, and bilirubin testing. Additionally, approximately 75 % of deaths occurred after the first 24 h of life, considered the critical period in neonatal care for death due to asphyxia. Thus, it is more likely that a considerable proportion of deaths after 24 h were due to neonatal infections; systematic preventive antibiotic treatment, in particular among the vulnerable <32 weeks gestational age group, might prevent a portion of these deaths.

This study contains two important policy implications. First, our programme results provide compelling evidence that implementing specialised neonatal care for premature babies is possible at the district level in a low-income setting. Even for very low birth weight premature babies (<1500 g), acceptable survival rates could be achieved. While the programme was managed by MSF, it was run with limited technical resources, relying on high quality, on-site training of non-specialist staff and clear protocols for case management. We encourage other actors and national ministries of health to consider taking up this model at district level in similar settings. This model has also been strongly suggested in the recent publication, Born too Soon [15].

Second, based on our experience in Kabezi District Hospital, we suggest that integration of neonatal and obstetric care is an essential component of quality care for premature infants. A strong referral system for women in danger of premature delivery is of particular importance, in order to capture all premature neonates at a specialized facility in a timely fashion. Additionally, we recommend an integrated package of neonatal intensive care (NICU) and KMC.

In conclusion, this study shows the feasibility of providing specialized neonatal care for premature neonates at a district level in Africa. Good outcomes were achieved with limited resources, suggesting that the model of neonatal care in Kabezi District Hospital provides a way forward to reduce neonatal, and ultimately, paediatric mortality rates in low-income settings.

## Abbreviations

NICU: Neonatal Intensive Care Unit
KMC: Kangaroo Mother Care
MSF: Médecins sans Frontières
EOC: Emergency Obstetric Care
DFID: Department for International Development
UK: United Kingdom
LuxOR: Luxembourg Operational Research Unit
APGAR score: Appearance, Pulse, Grimace, Activity, and Respiration
STROBE: Strengthening the Reporting of Observational studies in Epidemiology
WHO: World Health Organization
SORT-IT programme: Structured Operational Research and Training Initiative
